# Evolution of the Knowledge Mapping of Climate Change Communication Research: Basic Status, Research Hotspots, and Prospects

**DOI:** 10.3390/ijerph191811305

**Published:** 2022-09-08

**Authors:** Meifen Wu, Ruyin Long, Shuhan Yang, Xinru Wang, Hong Chen

**Affiliations:** 1 School of Economics and Management, China University of Mining and Technology, Xuzhou 221116, China; 2 School of Business, Jiangnan University, Wuxi 214122, China; 3 The Institute for Jiangnan Culture, Jiangnan University, Wuxi 214122, China; 4 Institute for National Security and Green Development, Jiangnan University, Wuxi 214122, China

**Keywords:** climate change communication, literature review, CiteSpace, knowledge mapping

## Abstract

Climate change communication is a crucial strategy for addressing the major challenges of climate change, and the knowledge mapping analysis and overview of it helps to clarify research progress. Based on CiteSpace, 428 pieces of domestic and foreign literature are collected to clarify the basic status of climate change communication research and summarize research hotspots and prospects. The study found that: (1) The earliest traceable English literature on climate change communication appeared in 2000. The number of articles published has risen steadily since 2008, reaching its first peak in 2015. (2) In contrast, research into Chinese climate change communication began late and progressed slowly. The Chinese literature on climate change communication first appeared in 2009. Although domestic research generally continues to pay attention to this phenomenon, there is still room for development compared with international research. (3) The research hotspots for climate change communication are obtained through keyword co-occurrence analysis. Public perceptions of climate change are strongly influenced by political ideology. Since climate change has political attributes, people from different political parties or groups form their views on climate change through identity protection. (4) The research content on climate change communication can be summarized into the following six aspects: the development of climate change communication research; differences in public perceptions of climate change; factors influencing climate change communication; key elements of the climate change communication process; the important role of the media in climate change communication; and effective strategies for climate change communication. Finally, the shortcomings of this study are summarized and future research prospects on climate change communication are put forward from the perspectives of research methods, research contexts, and research paradigms.

## 1. Introduction

In the middle and late 1980s, climate change first appeared on the public agenda. In recent years, it has become an important global issue with the emergence of the greenhouse effect and the frequency of extreme weather [1]. As a social communication activity, climate change communication not only serves as a vital tool for influencing public opinion to advance understanding of climate change issues, but also as a channel for fostering effective government action on climate change [2]. Climate governance requires not only top-level design, but also public understanding, support, and action. Climate change communication is closely related to taking part in global climate governance and creating a community of human destiny. Therefore, a systematic review of climate change communication research at home and abroad and an analysis of the characteristics and attributes of its knowledge evolution can help to grasp the research progress and is of great significance for encouraging further research in this field.

Since climate change communication originated in western countries, the research at home and abroad is different. Regarding reviews of climate change communication, the majority of them currently use the qualitative analysis method. For example, Moser (2010) reviewed the history of climate change communication and discussed the challenges it faced [1]. By concentrating on reviews related to climate change communication since 2010, Moser (2016) summarized the significant progress, emerging trends, and research topics in this area, and described the essential requirements and opportunities in the future [3]. Few systematic reviews of climate change communication have been conducted through quantitative research methods such as econometric analysis using CiteSpace software. Quantitative analysis can incorporate a large amount of literature related to a field into analysis to make up for the lack of comprehensive samples in qualitative analysis. Therefore, in this study, CiteSpace is used to make a quantitative visual analysis of the research literature on climate change communication, reveal the research status of climate change communication at home and abroad, and predict future research directions. Due to the interdisciplinary nature of climate change communication, scholars from different professional backgrounds have studied climate change communication from different starting points. This study aims to help researchers and related practitioners have a deeper understanding of the current status and trends of climate change communication research, identify key literature, summarize research hotspots and main research contents, and form a panoramic knowledge network structure.

To clarify the current status of climate change communication research, we comb the existing research through a combination of quantitative and qualitative reviews. The following research questions are addressed in this study: (1) How has climate change communication research changed and evolved in China and other foreign countries? (2) What are the research hotspots in the field of climate change communication? (3) What are the main research contents of climate change communication? It is mainly analyzed from the following three aspects: First, the knowledge mapping of the field of climate change communication research is created using the bibliometric method, and a general overview of the field is presented from a global perspective. Second, to serve as a reference for a thorough analysis of the multi-level research trajectory of climate change communication, the current research contents in this field are sorted out. Third, based on the literature review, the research prospects that can be further promoted in the future are presented, along with a summary of the shortcomings of the current research, which serves as a useful guide for encouraging the integration of domestic research with the global frontier.

The rest of this paper is organized as follows. Section 2 examines the comparison of concepts related to climate change communication and their conceptual connotations. Section 3 provides details about research methods and literature collection, mainly about the introduction of CiteSpace and data collection. Section 4 presents the results of the bibliometric analysis and visualization of knowledge mapping. The last section summarizes the research conclusions and puts forward future research prospects.

## 2. Climate Change Communication

### 2.1. Concepts Related to Climate Change Communication

According to Wang (2018), climate change communication research as a whole started late and has absorbed the characteristics of related research in the development process, mainly including environmental communication, development communication, health communication, science communication, risk communication, and political communication [4].

This also reflects the professionalism of climate change issues and their gradual expansion. Similarly, Moser (2010) argues that much of the information and assumptions about climate change communication have evolved from other fields [1]. To sort out the research progress of climate change communication, it is first necessary to compare communication on topics such as environmental communication, risk communication, and health communication (Table 1).

Risk communication, scientific communication, environmental communication, and other research related to climate change communication all emphasize “one-way dispersion” to “equal dialogue” [10]. Since climate change is characterized by a lack of visibility and immediacy, delay or lack of sense of achievement in taking action, the contest between cognitive limitations and technological progress, and the complexity and uncertainty of climate change [1], climate change communication is more challenging than environment, crisis, or health problem communication.

### 2.2. The Conceptual Connotation of Climate Change Communication

Due to the short history of climate change communication, there is no consensus in the academic community on the definition of climate change communication. The Climate Communication Project of Yale University proposes that climate change communication should achieve two major goals: one is to improve the public’s understanding and participation in climate change science and solutions. Second, the general public, government leaders, enterprises, academia, and the media are urged to jointly cope with climate change through climate change communication. The European Space Program’s Climate Change Project believes that the goal of climate change communication research is to make more people aware of climate change, enhance the public’s crisis awareness of climate change, improve the public’s sense of responsibility for adapting to and mitigating the impact of climate change, and provide best practice suggestions and examples for adapting to climate change and reducing emissions. According to the American scholar Priest (2019), climate change communication is a type of mass communication, which is classified by the research object [11].

Professor Zheng was the first scholar to define climate change communication in China, and this definition is also the most widely used one at present: climate change communication is a kind of communication activity, which aims to understand and master climate change information and related scientific knowledge for society and the public, raise the public’s awareness of climate change, enhance their sense of crisis, and adapt to the sense of responsibility of mitigating the impact of climate change, and seek solutions to climate change problems through changes in public attitudes and behaviors [12]. This definition not only clarifies the mass communication nature of climate change communication, but also emphasizes the goal of this communication activity [13].

## 3. Research Methods and Literature Collection

### 3.1. Research Methods

CiteSpace is a visual data analysis software used to help analyze trends in knowledge domains, and it presents the characteristics of nodes through two key metrics: frequency of occurrence and intermediary centrality. The frequency of occurrence reflects the number of times the node appears in the network, and the centrality of the intermediary reflects the importance of the node in the network [14]. It can statistically analyze the information of authors, keywords, and cited literature related to a certain knowledge area, and present the analysis results through visualization. Compared with systematic evaluation (meso-level) and meta-analysis (micro-level), the biggest advantage of this method is that it can visually analyze big data samples from a macro level, and help researchers understand the development trend of a knowledge field from an overall perspective [15]. The version of CiteSpace software used in this study is 5.6.R5. First, journals and keywords are selected as nodes to generate the map. The time zone chosen is from 2000–2022, one year equals one time zone segment, and the threshold is set at the top 50. Pathfinder + Pruning Sliced Networks is selected as the pruning method to simplify the network and highlight the key nodes. Other options are set according to the default settings of the software, and keyword cluster analysis and literature co-citation analysis are further carried out.

### 3.2. Literature Collection

Since climate change communication is expressed as “climate communication” in some contexts, this paper uses “climate change communication” and “climate communication” as search keywords for bibliometric analysis. The research data include Chinese and English literature, among which the English literature is collected from the Science Citation Index Expanded (SCI-EXPANDED) and the Social Sciences Citation Index (SSCI), index databases in the Web of Science core collection. The search time is set from 1985 to 2022, and a total of 387 English literature records are obtained. Papers or reviews are selected for literature types, the duplicates are removed, and, finally, 370 literature records are obtained. The Chinese literature is selected from the CSSCI and CSCD databases of CNKI, and the time is set from 1915 to 2022. After screening for irrelevant literature, 58 Chinese literature records are obtained.

## 4. Analysis of Bibliometric Results

### 4.1. Basic Distribution of Literature

#### 4.1.1. Analysis of the Number of Articles Published

**Figure 1:**
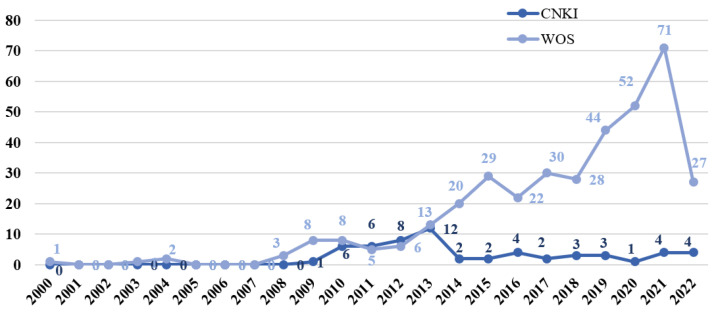
Trends in the publication of domestic and international literature on climate change communication.

The annual distribution of relevant domestic and international literature is shown in Figure 1. The earliest official climate change communication literature in English dates to 2000, according to the number of articles published in the WOS database. However, between 2001 and 2007, there were no studies related to climate change communication, except in 2004. The number of papers published has risen steadily since 2008, reaching its first peak in 2015. The Paris Climate Change Conference was held at the end of this year, and it is a landmark conference in the climate governance process, after the Copenhagen Conference in 2009, and it has attracted a high degree of attention [16]. After that, the number of articles published generally showed a steady growth trend, and the number of articles published in 2021 reached the highest value in recent years, amounting to 71 articles. 2021 is the year in which the Paris Agreement was fully implemented, which may be one of the important reasons for the rapid increase in the number of publications. In contrast, the research on climate change communication in Chinese started late and the number of articles published was relatively small, mainly because the research on climate change communication in China started after the financial crisis in 2008. However, due to the strong economic stimulus measures of the Chinese government in 2009, the Chinese public did not feel the economic crisis at that time, so the research on public opinion on climate change in China was not involved [17]. The Chinese literature on climate change communication first appeared in 2009, and then maintained a steady rise, reaching a peak in 2013 and then declining, maintaining an average of two articles per year from 2014 to the present, indicating that climate change communication research in China has developed relatively slowly. Although domestic research in general continues to focus on this phenomenon, there is still room for development compared with international research.

#### 4.1.2. Analysis of Core Journals

**Figure 2:**
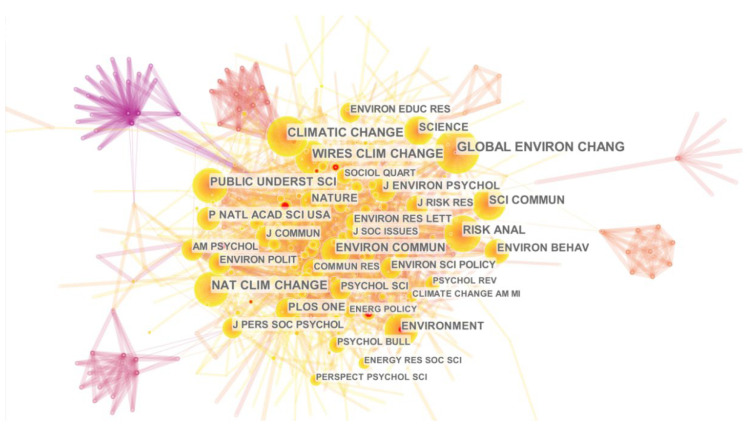
Journal knowledge map.

Core journals refer to important journals with a high number of citations [18]. As shown in Figure 2 and Table 2, Global Environmental Change, Climatic Change, Nature Climate Change, and WIREs Climate Change are the most highly cited journals in terms of the number of publications. In terms of impact, the American Sociological Review, Journal of Personality and Social Psychology, American Psychologist, and Environmental Research Letters are among the best. The findings help researchers quickly find journals that are relevant to their research topics.

### 4.2. Literature Visualization Analysis

#### 4.2.1. Keyword Analysis

Keyword analysis helps to gain an intuitive understanding of research hotspots and their evolution, and to predict future directions. A first impression of the topic overview of research hotspots in the target research area can be obtained from the co-occurrence of keywords and their clustering by CiteSpace. Since the amount of Chinese literature accounts for less than one-sixth of the overall sample and can be read one by one, this paper focuses on the knowledge graph analysis of the 370 collected English literature papers and a qualitative review, alongside the key Chinese literature.

After combining keywords with repetitive keywords such as “perception” and “risk perception”, the keyword frequencies and intermediary centrality were counted and ranked, and the top 36 keywords in terms of frequency were analyzed. Aside from the closely related words “climate change” and “climate change communication,” other high-frequency keywords include not only the related words of influencing factors, such as risk perception, attitude, and emotion, but also the related words of climate change communication post-variables, such as climate change adaptation and impact. The distribution of the above-mentioned high-frequency words indicates that there is much research on risk perception and adaptation to climate change in the research of climate change communication, and the research on psychological factors affecting climate change communication is still in the development stage. Sapiains Arrué and Ugarte Caviedes (2017) found through their research that the contribution of psychology to the challenge of climate change improves our understanding of society and climate change. Furthermore, psychological studies need to be part of wider multidisciplinary teams and work at multiple levels [19].

#### 4.2.2. Keyword Clustering Analysis

**Figure 3:**
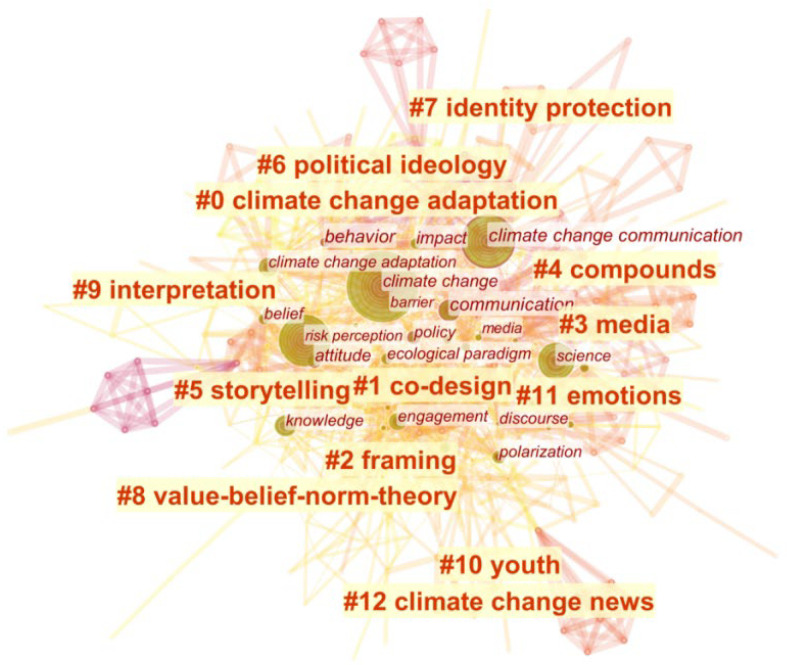
Keyword clustering map.

Clustering analysis integrates the connected nodes and extracts keywords, which helps to understand the contents of the map more intuitively. With the keyword co-occurrence analysis, the knowledge mapping network was optimized using the Pathfinder + Prunning sliced networks algorithm by slicing every 1 year from 2000 to2022, and 449 nodes and 1425 connected lines of the knowledge network of climate change communication research were obtained (as shown in Figure 3).

Combined with the keyword clustering map, it can be seen that the research hotspots of climate change communication are centered on #12 climate change news, #8 value–belief–norm theory is one of the most commonly used research theories, and #3 media is a channel for climate change communication. Climate change-related issues can be interpreted (#9 interpretation) by #1 co-design, #2 framing, creation of #4 compound words related to climate change (#4 compounds), and #5 storytelling. The purpose of climate change communication is to mobilize individual citizens, communities, commercial organizations, governments, and non-governmental organizations to participate in the action to deal with climate change, so that the public can adapt to climate change (#0 climate change adaptation), to achieve the goal of solving climate change problems. #6 Political ideology is a strong predictor of public opinion on climate change [20]. As climate change has a political nature, people from different political parties or groups form their climate change views through #7 identity protection. Additionally, young groups (#10 youth) are playing an increasingly significant role in climate change communication, as they are uniquely positioned to deal with the reality of climate change. #11 Emotions are one of the crucial psychological factors in communicating about climate change, and some emotions can inhibit actions [21].

National and international legislation to mitigate climate change usually takes ten years as a time cycle, and many decarbonization targets are concentrated in the period 2020–2050 [22]. The younger generation may be best suited to define the long-term social response to climate change, as they are the generation whose adult lives most closely overlap with this policy window. One could argue that the youth benefit and suffer most from climate change. Although they have been studying how to more effectively communicate climate change to the public for decades, people know very little about how young people can engage in an issue that will influence and define their generation. Corner et al. (2015) used survey data and qualitative research to examine young people’s awareness, concern, and doubts about climate change [23]. According to a nationally representative survey, Americans aged 18–35 are the most likely to ignore the negative effects of climate change, with only 21% believing that people are currently experiencing any negative effects [24].

Due to the invisibility and complexity of climate change, as well as the lack of intuitive experience for most people, it is necessary to disseminate the knowledge gained through experiments to the public to make the research results visible and relevant to daily life. For example, Dulic et al. (2016) used digital media as an immersive and experiential interface in their research to help communities adopt a more sustainable way of life. It involves participants in the informed decision-making process through dialogue and consultation at all levels of research, to provide an experiential environment for a comprehensive understanding of local climate change and create space for exploring complex challenges and solutions to climate change [21]. Additionally, climate change impact maps were co-designed and tested with local adaptation planning practitioners and experts in the climate change communication field, which provides important insights into the needs of adaptation planners and conveys the visual impact of climate change to adaptation policymakers [25]. Climate change communication is related to linguistics and rhetoric. To some extent, the carbon compound was once invented as a language framework in the debate on climate change and, to some extent, it acted like a popular metaphor once invented and spread to the public domain. Nerlich and Koteyko (2009) discussed climate change from a linguistic perspective. The research focused on the so-called “carbon compounds”, that is, vocabulary compounds centered on carbon (such as carbon footprint, carbon finance, carbon trading, carbon tax, etc.) to test how they play a role in climate change discourse. Linguistic analysis of the vocabulary and discourse composition around these “carbon compound words” is helpful to gain insight into the public. For example, carbon footprints, the media, NGOs, and governments are providing advice on how to reduce individuals’ “carbon footprint” almost every day as part of efforts to mitigate the impact of climate change [26].

However, not all the people who are concerned about climate change take corresponding actions to deal with climate change. Studies have shown that individuals who are highly concerned about climate change rarely take action to influence public policies. To evaluate the social psychology and cognitive drivers of climate action, Doherty and Webler (2016) developed a behavior model that integrates social cognitive theory, social norm research, and value–belief–norm theory. The results show that the communication strategy for frightened individuals and their public behavior should include strategies that foster positive descriptive social norms and effectiveness, which can motivate frightened individuals to participate in public actions [27]. The value–belief–norm theory is a robust theory that is the predictor of climate action, and it successfully explains individuals’ private environmental behavior. The value–belief–norm theory believes that individual norms drive behavior, and these norms are motivated by values, ecological world outlook, awareness of the consequences of environmental problems, and a sense of responsibility for problems [28,29]. Moreover, skillful use of image communication and visual communication methods, such as experiential scene reporting, the use of metaphors and categories, and narrative techniques in storytelling, are all effective communication methods that have been proved by practice.

Many scholars have explored this attribute, since climate change is not only a scientific issue, but also has a strong political undertone. For example, Hart and Nisbet (2012) used motivated reasoning, social identity, and persuasion theory in their study to examine how science-based messages increased public polarization on controversial scientific issues (e.g., climate change) and how the effects of messages about climate change were influenced by political partisanship and social identity. By letting 240 adults watch a simulated news story about the possible effects of climate change on the health of different groups, it is found that the impact on the identification of potential victims depends on the political parties of the participants. This partisanship increased the political polarization level in support of climate mitigation policies. Embedded social identity cues interacted with political orientation to amplify public polarization on the controversial scientific issue of climate change [30]. When there are opposing views in the group, and the biased process of protecting identity takes place, the position on policy-relevant facts becomes a marker of one’s group membership. For those who seek to communicate scientific consensus, a core challenge is to break the reinforcement cycle generated when viewers choose information channels that can both trigger identity protection and selectively distort scientific records [31,32]. Continued selective exposure may increase polarization [33], unless scientists and journalists explain the scientific consensus in places that appeal to conservatives and in ways that do not invoke identity protection [34]. Science communicators should create an information environment that is conducive to providing scientific evidence, without abandoning the values that are vital to one’s community [35]. To do this, it is necessary to reduce or get rid of hints to draw on scientific evidence through identity protection filters [36].

#### 4.2.3. Literature Co-Citation Analysis

**Figure 4:**
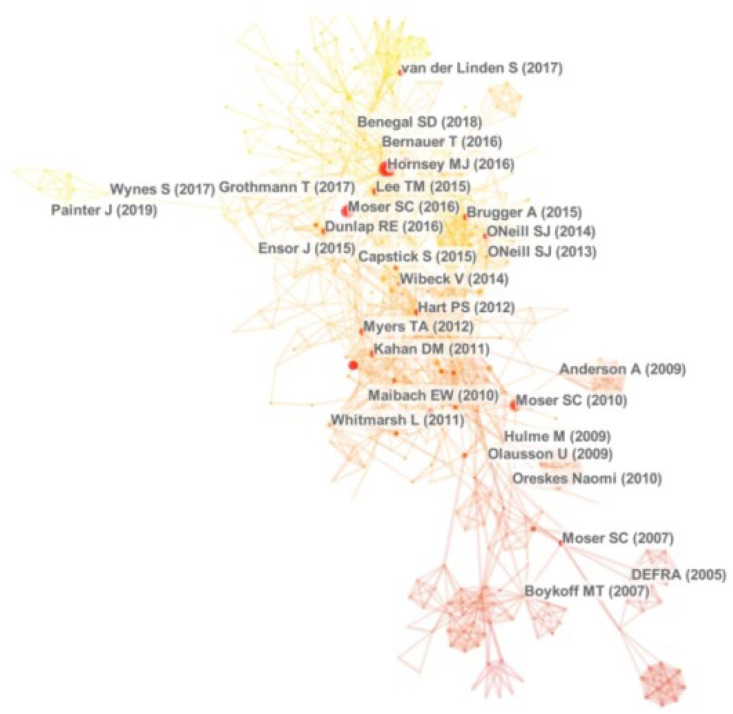
Literature co-citation map.

When two articles are cited by the same article, they are in a co-citation relationship. Since CNKI does not support exporting literature citation data, co-citation analysis cannot be performed. Only WOS records are analyzed for co-citation, and the results are as shown in Figure 4. Literature co-citation analysis can be efficiently identified as the important knowledge base of the research area, i.e., the core classical literature, among a large amount of cited reference information. The top ten articles ranked in descending order of co-citation frequency or centrality are the knowledge base in the field, which helps researchers to gain a preliminary understanding of the core research results in this field (Table 3). 

We read 20 core classic articles and the top 20 cited articles in Chinese literature in detail. Through the analysis of related literature, the research on climate change communication has been increasing in recent years, and the research contents have been enriched and the research variables have been expanded. Combined with the content interpretation of the sample literature, the research knowledge base of climate change communication and the main research contents of the classic literature can be summarized in the following six aspects.

**(1)**  **The emergence of climate communication research**

Communication studies investigate interactions between scientists, the media, policymakers, and stakeholders. Due to global warming and the complexity of climate change, climate change communication has attracted attention. With the development of the times, climate change communication has changed from convincing people that climate change is happening to convincing people to take practical measures to deal with it [37]. The issue of climate change is a product of industrialization, which first appeared in western countries. As a result, research on climate change communication also began in western countries, especially in developed countries such as the United Kingdom and the United States [38]. Researchers in the fields of environmental, psychological, anthropological, sociological, cultural, political, geographic, journalistic, and communication have been working on climate change communication issues since the end of the last century and the beginning of this century, and it has become an increasingly popular topic of research [12]. Although environmental reporting in the Chinese media started in the 1990s, and related research started at that time as well [39], it was relatively late to discuss climate change issues from the perspective of communication. Chinese climate change communication research first came to the public’s attention at the Copenhagen Climate Conference in December 2009, which can be viewed as the beginning of climate change communication in China [12].

**(2)**  **Differences in public perception of climate change**

The core subject of climate change communication is the public. The public perception of climate change was one of the earliest topics to be explored. Concerns arose in the period around 2007 since the issue of climate change entered public consciousness in the 1980s, but surveys show a sharp decline in belief and concern in many developed countries, followed by concerns that have stabilized in some parts of the world since around 2010. The experience of abnormal weather and other events seems to have some influence on the public’s perception. It is worth noting the growing political polarization in the United States, and there is a clear partisan divide in views on global warming. Increasingly more Republicans question the validity of climate science and ignore the urgency of this issue. In contrast, a growing number of Democrats embrace climate science and express concern about the issue, with the percentage of Democrats who believe global warming poses a serious threat steadily increasing [20].

In addition, public perceptions of climate change vary from country to country and fluctuate over time. Between countries, the growth of skepticism appears to be much greater in parts of Europe, such as the United Kingdom, Australia, and the United States, than in other regions, such as sub-Saharan Africa and South America, where concerns about climate change tend to increase. The climate communication project implemented by Yale University School of Environment and Forestry, the global climate change project by George Mason University’s Center for Climate Communication and the Pew Research Center, a leading global research organization, and the Center for Research on Environmental Decisions at Columbia University all have conducted some research on climate change communication projects. At the same time, relevant research results have been released or published. The aim is to make the American public realize the importance of climate change communication knowledge and understand the impact of climate change on their lives [13]. In 2012, the China Climate Communication Project Center conducted the first survey on Chinese public perceptions of climate change and climate communication, that is the “Survey on Chinese Public Perceptions of Climate Change and Climate Communication”. The survey results show that the Chinese public has a high degree of recognition of the scientific findings on climate change [40]. However, according to a 2015 Pew Research Center survey, the United States and China, the two largest carbon emitters, are the two least sensitive to climate change, with only 15% of Chinese and 30% of Americans expressing concern about the potential for harm from climate change [41].

**(3)**  **Influencing factors of climate change communication**

Due to the complexity of climate change communication issues, there are distinct differences in public attitudes towards climate change in different countries. The reasons for these differences can be understood from the macro-level of social structure and the micro-level of individual psychological mechanisms. The social dimensions include scientific, political, and economic factors, and there are relations and tensions among them. The individual psychological dimensions involve demographic and psychosocial characteristics, as well as emotional factors, of which political ideology and values are the most significant. In addition, the media is an important channel to communicate climate change information, and factors such as climate change knowledge, climate change beliefs, and environmental values are also influencing factors of climate change communication. Through a meta-analysis synthesizing 25 public opinion surveys and 171 academic studies from 56 countries, Hornsey et al. (2016) explored 27 antecedent influencing factors of climate change, as well as posterior variables. They found that many intuitively attractive variables (such as education, gender, subjective knowledge, and experience with extreme weather events) were overshadowed in their predictive power by values, ideology, worldview, and political orientation [42]. Psychological factors regarding climate change communication have received more attention in recent years. For example, Myers et al. (2012) conducted a nationally representative online survey of American residents in December 2010, asking the subjects to read news articles about the unique framework of climate change and pay attention to emotional dimensions in the research. The results show that among all audience groups, the public health focus was most likely to elicit emotional responses consistent with support for climate change mitigation and adaptation. The findings also suggest that the National Security Framework may have an adverse effect on anger among an audience that is already skeptical or dismissive of climate change issues [43].

**(4)**  **Key elements of the climate change communication process**

**Figure 5:**
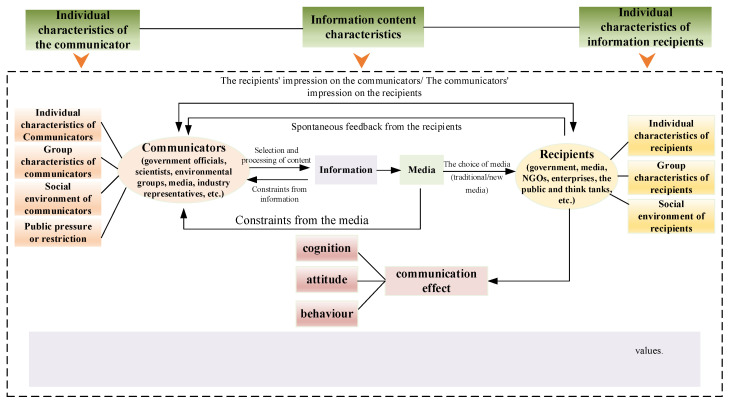
Key elements of climate change communication.

Communicating knowledge or information about climate change does not only deepen public education and improve scientific literacy, because people are not just lacking education, information, or understanding of climate change. The complexity of this problem lies in the fact that sociality has been intertwined with the use of scientific knowledge by both the general public and policymakers. Actionable knowledge and mechanisms can translate understanding and concern into action, but this process must be achieved through communication and support mechanisms [1]. Based on Moser’s (2010) study, the key elements of the climate change communication process were sorted out in terms of climate change communication purpose and scope, audience, framing, messages, communicators, communication modes and channels, and evaluation of results and communication effectiveness. The results are shown in Figure 5.

**(5)**  **The important role of the media in climate change communication**

The media is the main information source for the public [44]. Wilson (2000) believes that climate change knowledge in the United States mainly depends on the media, especially television. Although there are other sources of information, the public mostly uses the media as their primary information source [45]. In terms of the impact of different media on public beliefs about climate change, Krosnick and MacInnis (2010) found that viewers who regularly watched the Fox News Channel were less likely to accept scientists’ views on global warming than infrequent viewers [46]. By assessing the impact of different types of media, Feldman et al. (2012) investigated climate change coverage on three major cable news channels in their study and evaluated the relationship between the viewership of these channels and global warming beliefs. A content analysis of climate change coverage on the Fox News Channel, CNN, and MSNBC in 2007 and 2008 revealed that Fox was more dismissive of climate change than CNN and MSNBC. Further analysis showed that the relationship between cable news ratings (Fox and CNN/MSNBC) and global warming acceptance was stronger among Republicans than among Democrats [47].

With the rapid development of the Internet, people’s methods and habits for obtaining information are constantly changing. Scholars have also recognized the importance of new media in the process of communicating climate change information, and several studies on climate change communication in the new media environment have emerged. Blog posts are the raw data for studying users’ awareness, attitudes, and behavioral tendencies. Posts on new media such as Twitter can express part of users’ knowledge and opinions on issues such as climate change. Goritz et al. (2020) used an exponential random graph model based on Twitter data during climate change negotiations to compare the authority of investment promotion agencies with that of other actors. In the study, taking climate change policy as an example, an innovative method is proposed to measure and compare the authority in the digital field [48]. In addition, video is visual and intuitive, and the images have a strong impact, making it easy for people to form a basic judgment on an issue in a short period and spread it quickly. As the importance of online videos and video-sharing sites in the public’s daily life for accessing information is highlighted, how new media such as online videos construct and discuss important scientific issues such as climate change has attracted more and more attention from the public, especially researchers. The presentation of online video content and the expression of public opinion is becoming more important than ever before [2].

**(6)**  **Effective strategies for climate change communication**

To explore factors that predict climate change and effective messaging strategies, Scannell and Gifford (2013) surveyed 324 residents through an experimental approach and found that local attachment, local information frames, and gender were significant predictors of climate change participation [49]. Exploring effective strategies for climate change communication can be broadly divided into appeal and association strategies. (1) The two most common climate change communication strategies are fear appeal and emotional appeal. The manifestations of fear caused by climate change are widely used in the public sphere, such as TV pictures of melting glaciers, polluted rivers, endangered flora and fauna, smoking city streets, dirty urban villages, and so on. It shocks the audience and thus plays a warning role. However, different scholars have different perceptions about the impact of fear appeals on the effectiveness of climate change communication. O’neill and Nicholson-Cole (2009) showed in their study that fear may not be an appropriate tool for climate change communication, and that, although shocking, catastrophic, and large-scale depictions of climate change impacts may attract attention and concern, they do not inspire a sense of personal engagement with the issue, but may trigger barriers to participation [50]. On the other hand, people need emotional appeals because climate change communication is dealing with individuals who have unique spiritual lives [12]. (2) Since the costs of measures such as limiting CO_2_ emissions are current and the benefits are future, based on loss aversion theory and risk preferences, individuals’ aversion to costs and dissatisfaction with delayed benefits lead to a reluctance to take corresponding actions. Therefore, the relationship between climate change and the public’s personal experience should be established to enhance the public’s awareness of the importance of the climate change issue. This can be done by linking the energy consumption and carbon emissions of food, clothing, housing, and transportation to climate change, or by linking the invisible negative impacts of climate change to visible social issues or events, especially when extreme weather events occur, and communicators can make good use of this opportunity to transform abstract climate change into concrete and real issues for communication.

## 5. Research Conclusions and Prospects

### 5.1. Research Conclusions

In this study, the knowledge graph analysis method was used, and the CiteSpace visual analysis software was used to quantitatively analyze 370 documents in the Web of Science database. The basic characteristics of climate change communication were obtained through the analysis of the number of publications and core journals, and the research hotspots were obtained through the co-occurrence analysis of keywords. The main research contents of climate change communication were summarized through co-citation analysis and qualitative analysis of 58 documents in the CSSCI database of CNKI. The main research findings are as follows.

(1) In terms of the number of articles published in the WOS database, the earliest official climate change communication literature in English appeared in 2000. The number of articles published has gradually increased, reaching its first peak in 2015. After that, the number of articles has generally shown a steady growth trend, and the number of articles in 2021 reached the highest value in recent years. In contrast, Chinese climate change communication research started late, and the number of articles published is relatively small; the first Chinese literature on climate change communication appeared in 2009. Although domestic research in general continues to focus on this phenomenon, there is still much room for growth compared to international research.
(2) In terms of the number of publications, Global Environmental Change, Climatic Change, Nature Climate Change, and WIREs Climate Change are highly cited journals. In terms of impact, American Sociological Review, Journal of Personality and Social Psychology, American Psychologist, and Environmental Research Letters are among the top journals.
(3) Climate change communication research hotspots are centered around climate change news, and the value–belief–norm theory is one of the most commonly used research theories. The media is a channel for climate change communication. Climate change-related issues can be interpreted through co-design, frame setting, creating climate change-related compound words, and storytelling. Political ideology is a strong predictor of forming public climate change views. Since climate change has political attributes, people from different political parties or groups form their climate change views through identity protection. In addition, youth play an important role in climate change communication.
(4) The main research on climate change communication can be summarized in the following six aspects: the emergence of climate change communication research, public perception differences of climate change, factors influencing climate change communication, key elements of the climate change communication process, the important role of the media in climate change communication, and effective strategies for climate change communication.

### 5.2. Research Prospects

Through the above review of climate change communication research, research prospects for climate change communication are proposed from the perspectives of research content, research methods, etc.

(1) In terms of research content, the current research just describes and explores the current situation or phenomenon of climate change communication, but does not go deeper into it, and there are many shortcomings in research on the impact of climate change communication in terms of communication methods, communication channels, forms of information diffusion, and network structures. As Li (2013) mentioned in their study, although domestic and foreign institutions have conducted surveys specifically covering Chinese public perceptions of climate change, most of these surveys only examine public perceptions of climate change issues and related policies and actions, and rarely focus on the issues of channels, sources, contents, and strategies in climate change communication. Moreover, these surveys only stay at the level of data analysis, lacking in-depth theoretical analysis and innovative ideas [39]. In addition, the evaluation of communication effectiveness should be strengthened. Every information communication activity by the government costs a certain amount. Therefore, certain evaluation criteria should be used to judge whether the government’s information communication activities to address climate change are effective [51]. The evaluation of communication effects can be measured from cognitive, attitudinal, and behavioral aspects by adopting scientific indicators to construct evaluation methods according to different media, or based on the construction of a multi-modal fusion communication database of information, the characteristic expressions of each modality, such as gesture, expression, text, and voice, can be studied to complete the feedback of the effect of media content information and user interaction information, which can be fed into the design of media communication effectiveness improvement.
(2) In terms of research context, the information technology revolution and media iteration have brought about a new communication landscape, and the expression and interpretation of a specific media message have a profound impact on the effectiveness of that message and the audience’s perception. The means of information acquisition for generation Z have changed significantly from those of the previous generation. Mobile and visualization are the main trends in media consumption for generation Z. Instagram, Snapchat, and TikTok are three emerging social media platforms that are rapidly growing based on mobile visual information [52]. Communication technologies, such as the Internet, new media (e.g., blogs, Wikipedia, Twitter, computer games, and, especially, mobile media), and visual technologies have also made great strides. Therefore, in this new research context, applying machine learning and deep learning methods to mine data in different modalities in real media, building models to correlate and process multi-modal media data, and studying climate change communication in a multi-modal convergence perspective can help further promote public understanding of climate change and facilitate greater public participation in climate change action.
(3) In terms of research methods, framing is a very important communication choice [53], which can have a great impact on the persuasiveness of communication, public attitude change, trust, and participation. Some foreign scholars have focused on climate change communication strategies and techniques by using several empirical research methods, such as experimental methods, content analysis, and survey interviews, starting from disciplines such as communication, psychology, and public opinion [13]. The current development of computational social science, new media technology, and the advent of the era of big data has expanded the ideas of climate change communication research, and previously unavailable group data has gradually become possible. The use of big data, machine learning, network construction, and other methods to systematically and deeply study the relevant subjects and social operations in climate change communication is a major trend that cannot be ignored in future research in the field of climate change communication.
(4) In terms of research paradigms, future research must focus on how different communication approaches and strategies can encourage deeper personal concern and lifestyle engagement with climate change [51]. At present, whether it is environmental communication, science communication, or risk communication, all of them have begun to emphasize the transition from the “technical paradigm” of public understanding to the “democratic paradigm” that focuses on public participation. China’s climate change communication also needs more local, on-the-ground stories that are more relevant to the public. In this regard, it is necessary to activate user resources across the web to help popularize the production and communication of climate knowledge [54]. Since the new research context and paradigm may involve construal level theory, risk amplification theory, emotional contagion theory, planned behavior theory, etc., variables such as psychological distance and psychological barriers should be added, and network characteristics such as scale-free networks and complex networks should be used to do some exploration of climate change communication in combination with contagion or communication models. At the same time, it is necessary to follow the trend of the era of the knowledge economy and guide the whole society to participate in climate change communication.

## Figures and Tables

**Table 1:** Comparison of concepts related to climate change communication.

Related Concepts	Concept Connotation
Environmental communication	Any kind of communication practice and approach to the expression of environmental issues that aims to change the structure and discourse system of social communication [5].
Risk communication	Risk communication refers to the interactive process of exchanging information and opinions among individuals, groups, and institutions, which include natural or human-made hazards, such as flooding, wildfires, heatwaves, and droughts [6].
Health communication	Health communication is a social activity that uses various methods to promote and popularize health science and technology knowledge related to human physical and mental health, advocate health science methods, and communicate health science ideas [7].
Science communication	Science communication refers to the communication activity of popularizing scientific knowledge, promoting scientific ideas, and cultivating scientific spirit in the public through mass media [8].
Political communication	Political communication is the operation process of the organic system of political information diffusion, acceptance, identification, and internalization within and among political communities, and it is the flow process of political information within and among political communities [9].

**Table 2:** Highly cited journals.

Number	Cited Journals	Frequency	Cited Journals	Centrality
1	GLOBAL ENVIRONMENTAL CHANGE	267	AMERICAN SOCIOLOGICAL REVIEW	0.10
2	CLIMATIC CHANGE	236	JOURNAL OF PERSONALITY AND SOCIAL PSYCHOLOGY	0.09
3	NATURE CLIMATE CHANGE	206	AMERICAN PSYCHOLOGIST	0.09
4	WIRES CLIMATE CHANGE	190	ENVIRONMENTAL RESEARCH LETTERS	0.08
5	SCIENCE COMMUNICATION	159	PUBLIC OPINION QUARTERLY	0.08
6	RISK ANALYSIS	158	AMERICAN JOURNAL OF PREVENTIVE MEDICINE	0.08
7	PUBLIC UNDERSTANDING OF SCIENCE	152	AMERICAN JOURNAL OF SOCIOLOGY	0.08
8	ENVIRONMENTAL COMMUNICATION	136	COMMUNICATION RESEARCH	0.07
9	JOURNAL OF ENVIRONMENTAL PSYCHOLOGY	117	ECOLOGICAL ECONOMICS	0.07
10	PLOS ONE	114	ADVANCES IN EXPERIMENTAL SOCIAL PSYCHOLOGY	0.07
11	ENVIRONMENTS	113	ENVIRONMENT AND PLANNING A	0.07
12	PROCEEDINGS OF THE NATIONAL ACADEMY OF SCIENCE USA	112	COMMUNICATION THEORY	0.06
13	SCIENCE	107	CLIMATIC CHANGE	0.05
14	ENVIRONMENT AND BEHAVIOR	104	ENVIRONMENTAL POLITICS	0.05
15	NATURE	92	PSYCHOLOGICAL BULLETIN	0.05

**Table 3:** Top 10 co-cited articles by citation frequency or centrality.

Cited References (High-Frequency)		Cited References (High betweenness Centrality)	
Freq	Title	Centrality	Title
28	Meta-analyses of the determinants and outcomes of belief in climate change 10.1038/NCLIMATE2943	0.22	Reorienting climate change communication for effective mitigation: forcing people to be green or fostering grass-roots engagement? 10.1177/1075547008328969
25	Reflections on climate change communication research and practice in the second decade of the 21st century: what more is there to say? 10.1002/wcc.403	0.17	Boomerang effects in science communication: How motivated reasoning and identity cues amplify opinion polarization about climate mitigation policies 10.1177/0093650211416646
17	A public health frame arouses hopeful emotions about climate change 10.1007/s10584-012-0513-6	0.14	The climate on cable: The nature and impact of global warming coverage on Fox News, CNN, and MSNBC 10.1177/1940161211425410
16	Communicating climate change: history, challenges, process and future directions 10.1002/wcc.11	0.11	The polarizing impact of science literacy and numeracy on perceived climate change risks 10.1038/NCLIMATE1547
14	The polarizing impact of science literacy and numeracy on perceived climate change risks 10.1038/NCLIMATE1547	0.11	Communicating climate change: Why frames matter for public engagement 10.3200/ENVT.51.2.12-23
14	Cultural cognition of scientific consensus 10.1080/13669877.2010.511246	0.11	The potential of microblogs for the study of public perceptions of climate change 10.1002/wcc.273
14	Personally relevant climate change: The role of place attachment and local versus global message framing in engagement 10.1177/0013916511421196	0.09	Meta-analyses of the determinants and outcomes of belief in climate change 10.1038/NCLIMATE2943
14	Boomerang effects in science communication: How motivated reasoning and identity cues amplify opinion polarization about climate mitigation policies 10.1177/0093650211416646	0.09	Consensus on consensus: a synthesis of consensus estimates on human-caused global warming 10.1088/1748-9326/11/4/048002
13	The political divide on climate change: Partisan polarization widens in the US 10.1080/00139157.2016.1208995	0.08	International trends in public perceptions of climate change over the past quarter century 10.1002/wcc.321
13	“Fear won’t do it” promoting positive engagement with climate change through visual and iconic representations 10.1177/1075547008329201	0.07	Social norms and efficacy beliefs drive the alarmed segment’s public-sphere climate actions 10.1038/NCLIMATE3025

## Data Availability

Not applicable.
